# Germline mutations in retinoma patients: Relevance to low-penetrance and low-expressivity molecular basis

**Published:** 2009-04-17

**Authors:** Hana Abouzeid, Daniel F. Schorderet, Aubin Balmer, Francis L. Munier

**Affiliations:** 1 Jules-Gonin Eye Hospital, Lausanne, Switzerland; 2 Institut de Recherche en Ophtalmologie (IRO), Sion, Switzerland; 3 Ecole Polytechnique Fédérale de Lausanne (EPFL), Lausanne, Switzerland

## Abstract

**Purpose:**

To study phenotype-genotype correlation in patients who have retinoma, which is a benign tumor resembling the post irradiation regression pattern of retinoblastoma (RB).

**Methods:**

We selected patients who had retinoma and positive family history for RB and patients who had retinoma in one eye and either retinoma or RB in the other eye. The study included 22 patients with available DNA: 18 from 11 families and four sporadic cases. DNA was extracted from peripheral blood leukocytes. The *RB1* gene was screened by DHPLC and direct sequencing of the promoter and all the exons.

**Results:**

We identified 17 occurrences of 11 distinct germline mutations in two sporadic and in 15 familial cases (nine families). The 11 identified mutations were located in exons 1, 10,11,13,14, and 19 to 23. Four of the identified mutations were not previously reported, including g.64407delT, g.153236A>T, g.156743delTCTG, and g.162078delA. Eight out the 11 mutations were truncating and three were nontruncating (missense). There was no correlation between the type of mutation and the number of tumor foci per eye (RB or retinomas). Highly heterogeneous intrafamilial expressivity was observed.

**Conclusions:**

To our knowledge, this study is the largest series of mutations of consecutive retinoma patients. The present data suggest that the type of inherited mutations underlying retinoma is undistinguishable from RB related ones, i.e., largely dominated by truncating mutants. This finding is in contrast with the *RB1* genotypic spectrum of mutations associated with low-penetrance RB, i.e., nontruncating mutants. The molecular mechanism underlying low-penetrance and attenuated expressivity (retinomas) appeared to be distinct.

## Introduction

Mutations of the *RB1* gene can result in either malignant retinoblastoma (RB; OMIM 180200) or benign retinoma [1]. Retinoma mimics the postirradiation regression pattern of RB [1-4]. On histology, necrosis and mitoses are absent in contrast with RB [5]. According to Knudson’s observation, both alleles of the *RB1* gene must be inactivated to develop a tumor [6]. Hereditary germline mutations account for 40% of cases and nonhereditary somatic mutations for the remaining 60% [7]. For hereditary cases, mode of inheritance is autosomal dominant; 90% of germline-mutation carriers develop RB or retinoma (high penetrance) with most of them presenting multiple tumors in both eyes (high expressivity). Nevertheless, low penetrance is observed when germline mutation carriers do not develop RB and reduced expressivity when only unilateral RB or retinoma occurs [8,9]. To better describe low-penetrance RB families with regard to both penetrance and expressivity, Lohmann et al. [9] introduced the disease-eye ratio (DER), which is the ratio of the number of eyes containing tumors to the number of mutation carriers in a family. Typically, diseased-eye ratios are less than 1.0 in low-penetrance families and 1.5 or greater in full-penetrance families [9].

In 70% of RB cases loss of the wild-type allele, or loss of heterozygosity happens through mitotic recombination or nondisjunction with or without concomitant duplication of the mutated chromosome [10]. If the predisposing mutation is truncating, one could expect that loss of heterozygosity would lead to complete loss of functional protein and thus to RB with high penetrance and expressivity [8,9,11,12]. In contrast, the molecular basis of low-penetrance RB is dominated by nontruncating mutations [8,9,11,12]. Functionally, the *RB1* mutations causing low-penetrance RB were shown either to reduce the level of expression of normal *RB1* protein or produce a mutant *RB1* protein that is only partially inactivated [8,9]. However, recent insights strongly call into question this assumption with the report of a chain-terminating mutation in *RB1* exon 1 in a large low-penetrance family with unilateral RB and retinoma [13].

In this report, we correlate the clinical features of 17 retinoma patients to their underlying *RB1* germline mutations. Results are discussed in light of the recent advances regarding the molecular basis of RB oncogenesis.

## Methods

The study was conducted in accordance to the tenets of the Declaration of Helsinki. Out of more than 500 patients with RB and their first-degree relatives treated between 1964 and 2008, we selected patients who had retinoma and a positive family history of RB or patients who had retinoma in one eye and either RB or retinoma in the other eye. Patients were referred to us either from university eye clinics and private Swiss practitioners or from neighboring European countries. All patients were examined and treated at the Retinoblastoma Clinic of the Jules-Gonin Eye Hospital, Lausanne, Switzerland. All patients and their first-degree relatives underwent full ophthalmic examination and were documented by fundus photographs and ultrasonography. Fluorescein and indocyanine angiography was performed in selected patients. Previously described diagnostic criteria for retinoma and phthisis were used to identify the retinoma subpopulation [1]. Eight patients for whom DNA was not available were excluded from this study. Informed consent was obtained from all patients or parents to draw blood and perform genetic analysis. A total of 22 patients were selected and screened for *RB1* mutations. Charts were reviewed for specific clinical features such as age at diagnosis, tumor type (RB/retinoma), and number of tumor foci per eye. The number of RB foci could not be determined in all cases except one, which had a diffuse form of RB, because patients had been enucleated elsewhere in their childhood and we did not have access to the clinical or histopathological data. DER was calculated for each family.

DNA was extracted from blood leukocytes and used for PCR amplification. Genetic analysis was performed at the Institut de Recherche en Ophtalmologie. Previously described denaturing high-performance liquid chromatography (DHPLC) [14] and sequencing were used. Amplification was performed in a thermal cycler (GeneAmp 9700; Applied Biosystems, Foster City, CA), in a total volume of 30 μl. Each polymerase chain reaction (PCR) contained 100 ng genomic DNA, 0.9 nanomoles of each primer, and 15 μl master mix 2X (Qiagen, Hombrechtikon, Switzerland), with or without betaine. Reactions were subjected to 35 cycles of 94 °C for 1 min, annealing at the specific temperature for 1 min, 72 °C for 1 min, and a final extension step at 72 °C for 10 min. Sequence of primers and PCR conditions are presented in Table 1. After PCR amplification, products were screened for mutations using DHPLC on a WAVE system (Transgenomic, Crewe, Cheshire, UK). Buffer contained 0.1 M triethylammonium acetate (TEAA, Transgenomic). Buffer B contained 0.1 M TEAA and 25% acetonitrile HPLC grade (Sigma-Aldrich, Suffolk, UK). The flow rate was set at 1.5 ml/min and the Buffer B gradient increased by 5% per minute for 2 min. The optimum temperature was determined by the Wavemaker software (Transgenomic) for each DNA fragment, and a time shift was applied as needed [11]. When multiple melting domains were established, each domain was analyzed at the appropriate temperature. Initial Buffer B concentrations and temperatures for each fragment are listed Table 1. PCR fragments displaying DHPLC abnormal retention times were further sequenced on both strands using ABI Dye Terminator, version 1 or 3, in a final reaction volume of 10 μl, and electrophoresed on a 3130XL ABI genetic analyzer (Applied Biosystems). Sequences were aligned using the Chromasversion 2.23 (Technelysium, Tewantin, Australia). Screening for large deletions was performed by haplotype analysis using *RB1* flanking microsatellites D13S161, S13S164, D13S153, D13S1307, and D13S273. One primer was fluorescently labeled, and the product was separated on an automated sequencer (ABI XL3100; Applied Biosystems).

**Table 1:** Primers and PCR conditions.

**Region**	**Primer**	**Sequence 5′-3′**	**Amplicon [bp]**	**Annealing [°C]**
promoter	PromF	CTGGACCCACGCCAGGTTTC	340	61
	PromR	GTTTTGGGCGGCATGACGCCTT		
RB exon 1	1F	CCGGTTTTTCTCAGGGGACGTTG	340	56.4
	1R	TTGGCCCCGCCCTACGCACAC		
RB exon 2	2F	CTATTGAAACAAGTATGTACTG	331	54.3
	2R	GGGTAATGGAATTATTATTAGC		
RB exon 3	3F	CAGTTTTAACATAGTATCCAG	281	52.8
	3R	AGCATTTCTCACTAATTCAC		
RB exon 4	4F	GTAGTGATTTGATGTAGAGC	305	55
	4R	CCCAGAATCTAATTGTGAAC		
RB exon 5	5F	GCATGAGAAAACTACTATGAC	194	54.3
	5R	CTAACCCTAACTATCAAGATG		
RB exon 6	6F	CACCCAAAAGATATATCTGG	222	54.3
	6R	ATTTAGTCCAAAGGAATGCC		
RB exon 7	7F	CCTGCGATTTTCTCTCATAC	256	55
	7R	ATGTTTGGTACCCACTAGAC		
RB exon 8	8F	AGTAGTAGAATGTTACCAAG	380	50.8
	8R	TACTGCAAAAGAGTTAGCAC		
RB exon 9	9F	TGCATTGTTCAAGAGTCAAG	222	56
	9R	AGTTAGACAATTATCCTCCC		
RB exon 10	10F	TCTTTAATGAAATCTGTGCC	291	56
	10R	GATATCTAAAGGTCACTAAG		
RB exon 11	11F	GAGACAACAGAAGCATTATAC	245	54.2
	11R	CGTGAACAAATCTGAAACAC		
RB exon 12	12F	GGCAGTGTATTTGAAGATAC	310	52.5
	12R	AACTACATGTTAGATAGGAG		
RB exon 13	13F	CTTATGTTCAGTAGTTGTGG	342	54.3
	13R	TATACGAACTGGAAAGATGC		
RB exon 14	14F	GTGATTTTCTAAAATAGCAGG	212	58.9
	14R	TGCCTTGACCTCCTGATCTG		
RB exon 15+16	15/16F	CAATGCTGACACAAATAAGG	366	55
	15/16R	AGCATTCCTTCTCCTTAACC		
RB exon 17	17F	AAAAATACCTAGCTCAAGGG	339	56
	17R	TGTTAAGAAACACCTCTCAC		
RB exon 18	18F	TGTACCTGGGAAAATTATGC	340	56.4
	18R	CTTTATTTGGGTCATGTACC		
RB exon 19	19F	ATAATCTGTGATTCTTAGCC	273	56
	19R	AAGAAACATGATTTGAACCC		
RB exon 20	20F	AAAGAGTGGTAGAAAAGAGG	335	56.4
	20R	CAGTTAACAAGTAAGTAGGG		
RB exon 21	21F	AAACCTTTCTTTTTTGAGGC	328	54
	21R	TACATAATAAGGTCAGACAG		
RB exon 22	22F	TAATATGTGCTTCTTACCAGTC	313	56
	22R	TTTAATGTTTTGGTGGACCC		
RB exon 23	23F	ATCTAATGTAATGGGTCCAC	287	54.2
	23R	CTTGGATCAAAATAATCCCC		
RB exon 24	24F	GAATATAGTTTGTCAGTGGTTC	273	52 53
	24R	GTGTTTGAATAACTGCATTTGG		
RB exon 25	25F	GGTTGCTAACTATGAAACAC	297	54.2 55
	25R	AGAAATTGGTATAAGCCAGG		
RB exon 26	26F	AGTAAGTCATCGAAAGCATC	209	52.8
	26R	AACGAAAAGACTTCTTGCAG		
RB exon 27	27F	CGCCATCAGTTTGACATGAG	237	54.2

## Results

Out of the 22 selected patients, 18 were familial from 11 families and four were sporadic. Out of the 18 familial cases, we identified mutations in 15 cases, which were from nine families, and we found mutations in two of the four sporadic cases. Thus, we had 11 index cases in total with proven germline mutations. Patient clinical features and mutation descriptions are detailed in Table 2.

**Table 2:** Clinical features and mutations description of 17 affected patients with hereditary retinoblastoma and/or retinoma harboring *RB1* mutations.

**Patient number**	**Family number**	**Age at diagnosis**	**Disease-eye ratio**	**Right eye foci**	**Left eye foci**	**Mutation location Exon**	**DNA Alteration**	**Protein Alteration**
1	F1	43 y	1.3	-	1 rc	1	g.2179_2183dupGGACC	L42RfsX25
2	F2	2y	1.1	2 rc	Rb	1	g.2196G>A	R46K
3	F2	4 y 2 m	1.1	-	1 rc	1	g.2196G>A	R46K
4	F2	62 y	1.1	-	1 rc	1	g.2196G>A	R46K
5	F3	33 y	1.7	4 rc	2 rc	10	g.64407delT *	348X
6	F4	5 y 8 m	1.8	3 rc	1 rc	11	g.65386C>T	R358X
7	F4	8 m	1.8	2 rc	Rb	11	g.65386C>T	R358X
8	F4	30 y 8 m	1.8	1 rc	1 rc	11	g.65386C>T	R358X
9	S	6 y 10 m	-	1 rc	1Rb	13	g.73843C>T	Q436X
10	F5	35 y 10 m	1.4	1 rc	1 rc	14	g.76430C>T	R445X
11	S	39 y	-	2 rc	1 rc**	19	g.153236A>T*	K615X
12	F6	1 year 6 m	2	Rb	1 rc	20	g.156743delTCTG *	675X
13	F7	32 y 2 m	2	1 rc	2 rc	21	g.160834G>C	E737D
14	F8	40 y 2 m	1.7	1 rc	1 rc	22	g.162078delA*	A766fsX44
15	F8	36 y 11 m	1.7	3 rc	1 rc	22	g.162078delA*	A766fsX44
16	F9	8 y 9 m	1.3	7 rc	5 rc	23	g.162237C>T	R787X
17	F9	1 y10 m	1.3	Rb	1 rc	23	g.162237C>T	R787X

All retinoma patients are presented in this table. All familial retinoma patients belong to families which are not low-penetrance ones with DER>1. There is no correlation between the number of tumor foci and the type of mutations. Identified mutations were distributed over several exons. Abbreviations: sporadic (S), familial (F), year (y), month (m), retinoblastoma (Rb), retinoma (rc), deletion (del), frameshift (fs), novel mutation (*), malignant transformation into retinoblastoma (**).

Median age at diagnosis was 30.8 years (range 8 months to 62 years). Mean number of retinoma foci per eye was 1.88±1.5. Four of the identified mutations were not previously reported (Table 2).

## Discussion

**Figure 1:**
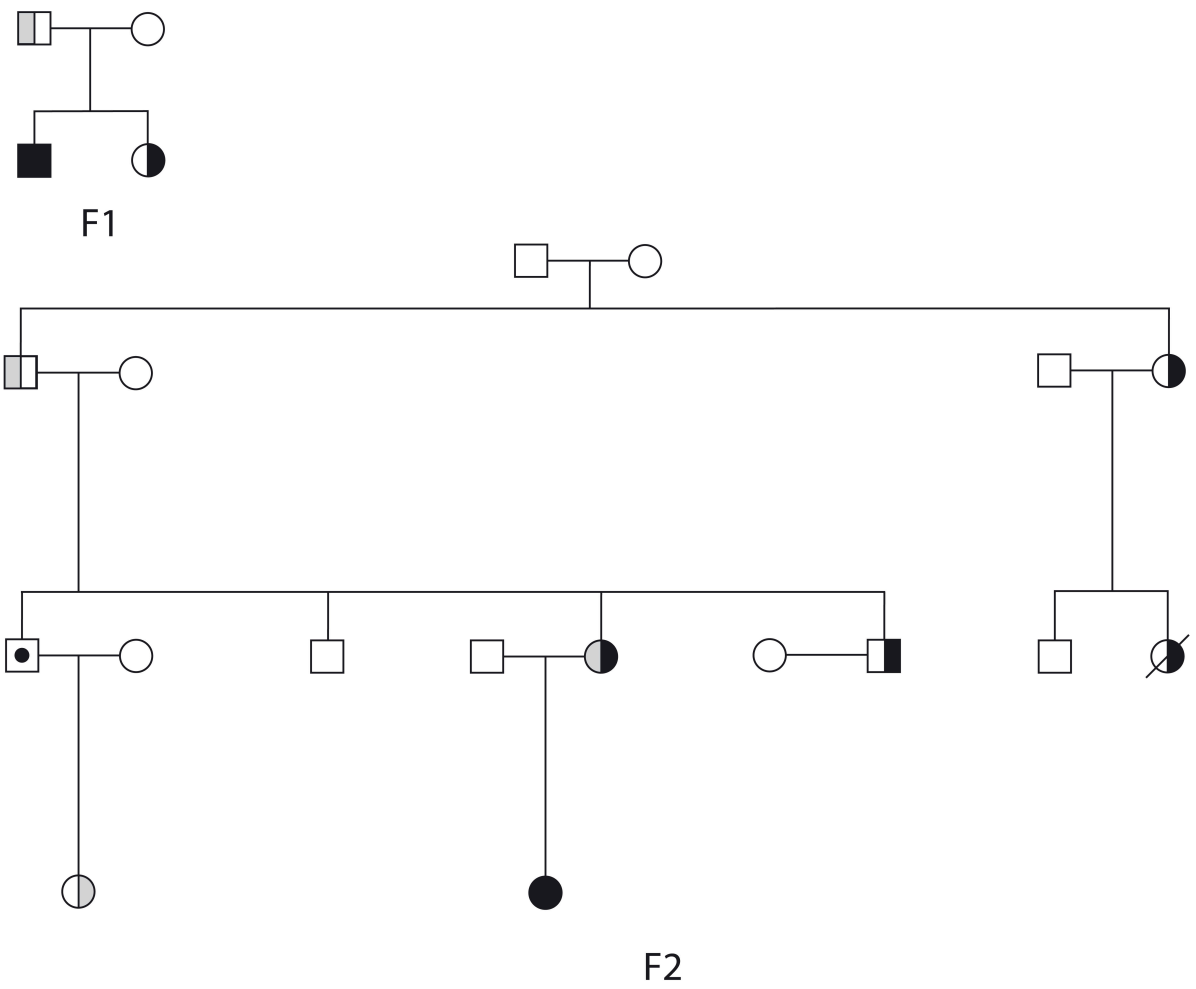
Pedigree of Family 1 and Family 2. Pedigree of the Family 1 in which the L42RfsX25 truncating mutation segregates is presented. Alternative translation might be the mechanism by which different level of expressivity are present within this family ranging from bilateral RB to unilateral retinomas. In Family 2 which includes an unaffected R46K mutation carrier, the level of expressivity is even lower. This mutation has been previously reported. Solid black symbols represent bilateral retinoblastoma (RB). Half black symbols represent unilateral RB. Solid gray symbols represent bilateral retinoma. Half gray symbols represent unilateral retinoma. The slashed symbol represents deceased individual, and symbol with a black dot represents an unaffected mutation carrier.

We studied 22 patients with retinomas and detected *RB1* mutations in 17 (15 familial cases and in 2 sporadic) of them. The 15 familial cases belonged to nine families that were not low-penetrance ones (DER 1.1 to 2). In accordance with the results of Sanchez-Sanchez et al. [13], the observation of retinoma with predisposing nonsense germline mutation does not support the hypothesis that truncating mutations do not cause retinoma. Indeed, truncating mutations were found in 70% of retinoma patients in this study. We tried to determine if a certain type of mutation could cause higher expressivity leading to multiple tumor foci in each eye, but there was no correlation between the type of mutation and the number of tumor foci per eye (RB or retinoma). In our cohort most mutations were either nonsense mutations, duplication or deletions. Although we did not analyze the consequences of the observed mutations at the RNA level, we can reasonably assume that they represent truncating mutations. The case of the two remaining mutations, R46K and E737D, is more complicated. They could represent true missense mutations or could affect splicing of the nearby intron. Their pathogenicity is not questionable, as they have been reported to cause bilateral RB [15]. We have previously described the L42RfsX25 mutation located in exon 1 [11]. Alternative translation might be the mechanism by which different levels of expressivity are present within this family (F1, Figure 1).

**Figure 2:**
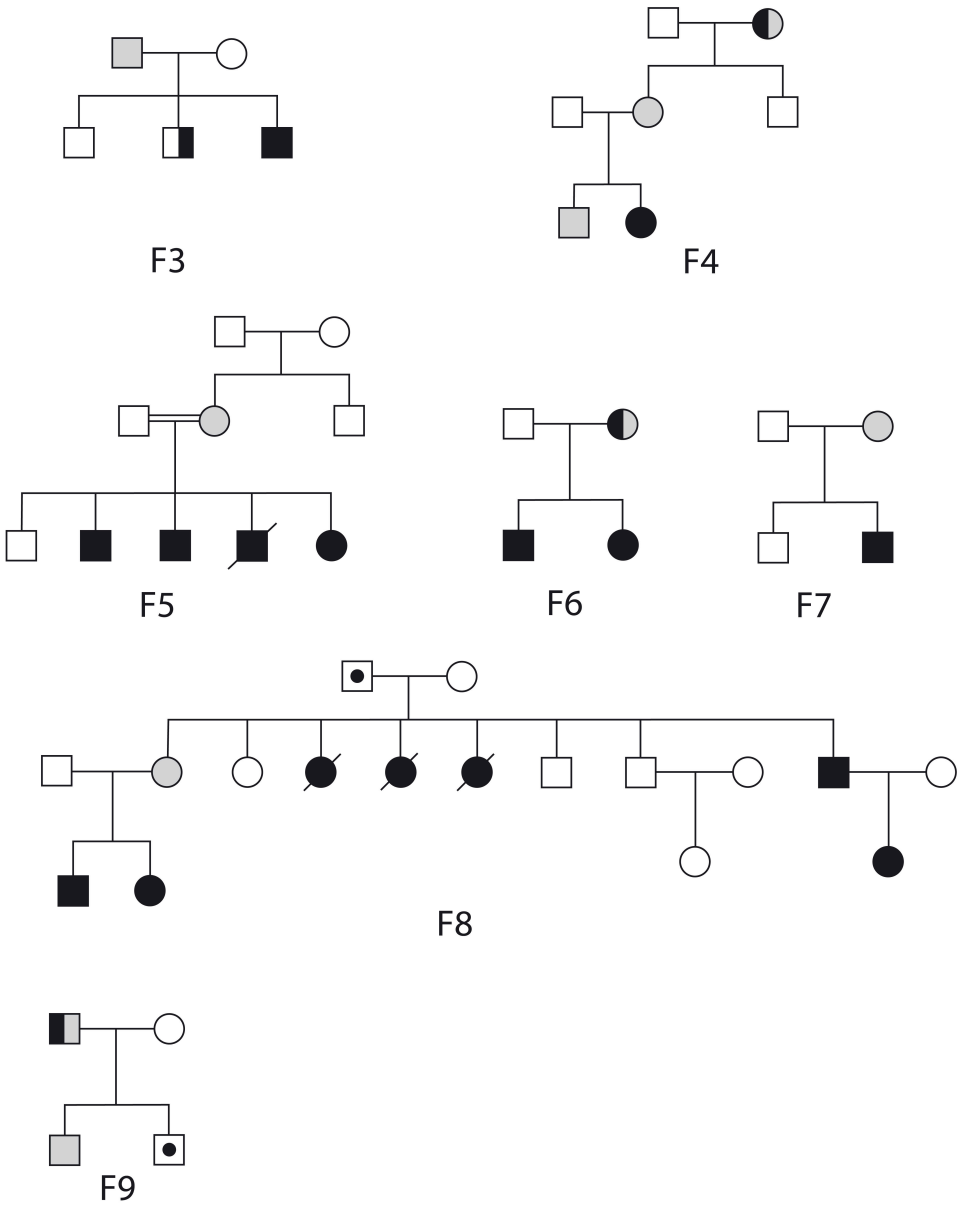
Pedigrees of Families 3 to 9. Seven families with retinomas family members in which *RB1* mutations have been identified, the families harbor different level of expressivity but are not low-penetrance ones. Causing mutations are all truncating except the E737D thus truncating mutations are responsible of the majority of retinomas in this study. In Families 6 and 9 novel *RB1* mutations segregate, namely the 675X and the A766fsX44 mutation. Solid black symbols represent bilateral retinoblastoma (RB). Half black symbols represent unilateral RB. Solid gray symbols represent bilateral retinoma. Half gray symbols represent unilateral retinoma. Slashed symbols represent deceased individuals, and symbols with black dot represent unaffected mutation carriers.

In contrast with the family studied by Sanchez-Sanchez [13], all nine families in our series had DER above 1.0, suggesting absence of low-penetrance. That the mean number of retinoma foci per eye was 1.88 indicates a relatively high level of expressivity. Furthermore, we observed that the same truncating mutation located in exon 11 and 23 led in Family 4 and 9 respectively (Figure 2) to highly heterogeneous expressivity ranging from the most severe bilateral RB to bilateral retinoma. This occurrence has not previously been described in association with severe truncating mutations. Notably, that the predisposing mutation was located at the carboxyl-terminus of the *RB1* gene (Family 9) suggests that another mechanism than alternative translation initiation may be involved. Unfortunately, in none of the families with truncating mutations could we perform functional analysis to determine the level of activity of the protein products. To the best of our knowledge, this is the first report focusing on germline mutations in retinoma cases. That we did not detect mutations in two families and two sporadic cases might be due to the technology applied that would not detect copy number changes of exons or most mosaicism. However, the 73% of *RB1* mutations (11 mutations found in 15 participants who had bilateral or familial RB) detected by the technologies applied suggests that the spectrum of mutations associated with retinoma is the same as for RB. Further work is needed in collaboration with other laboratories to use other mutation screening technologies to identify mutations in the remaining cases and to analyze the consequences of the observed mutations at the RNA level. This is beyond the intended scope of the present work. Although no statistical analysis was possible in this study, in accordance with other published series [4,16], we propose that retinoma should not be considered a form of attenuated expressivity [17-22], but rather the result of variable expressivity occurring in a penetrance independent manner.

Gallie et al. [23] have hypothesized that the stage of cell maturation at which the second *RB1* mutation occurs is determinant for the phenotype expression. We observed one case in this series, Patient 11, who underwent malignant transformation of his retinoma, at an adult age. This observation has been previously reported by others [2,4,23,24] and may support the stage of cell maturation theory with the understanding that a primitive cell could have remained latent for many years before becoming activated in the eye of an adult [23]. Another mechanism has been proposed to understand attenuated expressivity represented in this study by retinoma. Recent histopathological analysis of eyes enucleated for RB showed retinoma tumors adjacent to both normal retina and RB tumors in up to 15.6% (20/128), suggesting clonal progression from a normal cell to a benign one and finally to a malignant cell [16]. Other researchers have [16] reported molecular evidence of this clonal progression, showing that retinomas and RBs were homozygous null for *RB1*, could share the same mutation of whatever class, including stop codon, and that retinomas expressed senescence proteins maintaining them in an arrested state. Retinomas displayed genomic changes such as gain of oncogenes and genomic instability to a lesser degree than RB, which is thought to develop by escaping the senescence state of retinomas [16]. This hypothesis of increasing genomic instability for the development of RB is in accordance with the results we present. Unfortunately, we were not able to study the type of the second-hit mutation, which might be determinant, too, in cell destiny.

### Conclusions

RB development understanding remains a challenging but mandatory task due to the life-threatening complications it can induce. Its causative gene, *RB1,* has opened the way to the two-hit theory [6], the limitations of which have been highlighted by the study of retinoma, a benign tumor. For a long time, retinoma has been considered to be part of the low-expressivity as well as low-penetrance presentation and thought to be caused by less severe inherited mutations. We have shown in this series that even severe inherited mutations segregating in families with bilateral RB patients can also cause retinoma. Recently, it has been shown that RB emerges from retinoma after accumulation of genomic changes, whereas retinoma develops after homogeneous loss of *RB1* [16]. Thus, what we used to consider as low or attenuated expressivity should be revisited as a step in the cell pathway to malignant tumor development. Genetic counseling, treatment, and follow-up recommendations may highly be influenced by such advances in RB and retinoma development understanding.
